# Associations between *TUBB-WWOX* SNPs, their haplotypes, gene-gene, and gene-environment interactions and dyslipidemia

**DOI:** 10.18632/aging.202514

**Published:** 2021-02-17

**Authors:** Chun-Xiao Liu, Rui-Xing Yin, Zong-Hu Shi, Peng-Fei Zheng, Guo-Xiong Deng, Yao-Zong Guan, Bi-Liu Wei

**Affiliations:** 1 Department of Cardiology, Institute of Cardiovascular Diseases, The First Affiliated Hospital, Guangxi Medical University, Nanning 530021, Guangxi, People’s Republic of China; 2 Guangxi Key Laboratory Base of Precision Medicine in Cardio-Cerebrovascular Disease Control and Prevention, Nanning 530021, Guangxi, People’s Republic of China; 3 Guangxi Clinical Research Center for Cardio-Cerebrovascular Diseases, Nanning 530021, Guangxi, People’s Republic of China; 4 Department of Prevention and Health Care, The Fourth Affiliated Hospital, Guangxi Medical University, Liuzhou 545005, Guangxi, People’s Republic of China

**Keywords:** dyslipidemia, tubulin beta class I gene, WW domain-containing oxidoreductase gene, single nucleotide, interactions

## Abstract

In this study, we investigated associations between single nucleotide polymorphisms (SNPs) in the tubulin beta class I (*TUBB*) and WW domain-containing oxidoreductase (*WWOX*) genes, gene-gene interactions, and gene-environment interactions and dyslipidemia in the Chinese Maonan ethnic group. Four SNPs (rs3132584, rs3130685, rs2222896, and rs2548861) were genotyped in unrelated subjects with normal lipid levels (864) or dyslipidemia (1129). While 5.0% of Maonan subjects carried the rs3132584TT genotype, none of the Chinese Han in Beijing subjects did. Allele and genotype frequencies differed between the normal and dyslipidemia groups for three SNPs (rs3132584, rs3130685, and rs2222896). rs2222896G allele carriers in the normal group had higher low-density lipoprotein cholesterol and lower high-density lipoprotein cholesterol levels. The rs3132584GG, rs3130685CC+TT, and rs2222896GG genotypes as well as the rs2222896G-rs2548861G and rs2222896G-rs2548861T haplotypes were associated with an elevated risk of dyslipidemia; the rs2222896A-rs2548861T and rs2222896A-rs2548861G haplotypes were associated with a reduced risk of dyslipidemia. Among the thirteen *TUBB-WWOX* interaction types identified, rs3132584T-rs3130685T-rs2222896G-rs2548861T increased the risk of dyslipidemia 1.371-fold. Fourteen two- to four-locus optimal interactive models for SNP-SNP, haplotype-haplotype, gene-gene, and gene-environment interactions exhibited synergistic or contrasting effects on dyslipidemia. Finally, the interaction between rs3132584 and rs2222896 increased the risk of dyslipidemia 2.548-fold and predicted hypertension.

## INTRODUCTION

The average global prevalence of dyslipidemia is about 20% [1–3], and it is higher in patients with premature coronary arteriosclerotic diseases (CAD) [4] and chronic kidney disease [5]. Dyslipidemia plays crucial roles in the pathogenesis of hypertension, CAD, stroke, and chronic kidney failure that differ based on etiology, and identification of novel and safe treatments that decrease blood lipid levels, especially the level of low-density lipoprotein cholesterol (LDL-C), would be beneficial [6]. It is well known that lifestyle, environmental, and genetic factors all contribute to dyslipidemia. In the past decade, technological advances in biological sequencing technology have enabled genome-wide association studies (GWASs) that have helped thoroughly characterize risk factors for dyslipidemia.

The tubulin beta class I gene (*TUBB*, gene ID: 203068) located on chromosome 6 in humans encodes a beta tubulin protein that is an essential component of microtubules. Microtubules perform many cellular functions, including chromosome segregation, maintenance of cell shape, transport and motility, and organelle distribution [7]. As a substrate of peptidylarginine deiminases, *TUBB* participates in citrullination, which is related to growth, infiltration, and drug resistance in some tumor cells. Thus, mutations in *TUBB* are involved in complex diseases [8] and carcinoma [9]. The WW domain-containing oxidoreductase gene (*WWOX,* gene ID: 51741) on human chromosome 16 encodes a member of the short-chain dehydrogenases/reductases protein family that is involved in a variety of important cellular processes, including induction of apoptosis, cell development, and steroid metabolism [10, 11]. Mutations in *WWOX* are not only associated with multiple types of cancer [11], but also with lipid metabolism [12]. Both *TUBB* and *WWOX* are ubiquitously expressed in many tissues, including fat (reads per kilobase per million mapped reads (RPKM) 97.7 and 1.6, respectively), heart (RPKM 67.2 and 0.5) and liver (RPKM 58.1 and 0.9). Because of their functions and tissue distribution, single nucleotide polymorphisms in the *TUBB* and *WWOX* genes can have similar effects on lipid profiles. Recently, GWASs revealed that SNPs at locations rs3132584 and rs3130685 in *TUBB* are associated with LDL-C profile [13], while SNPs at rs2222896 and rs2548861 in *WWOX* are associated with LDL-C and high-density lipoprotein cholesterol (HDL-C) profiles [14], respectively. However, associations between *TUBB* and *WWOX* and lipid traits in the Chinese population have not been examined; we hypothesize that modifications of *TUBB* and *WWOX* affect blood lipid levels in this population as well.

The incidence of dyslipidemia in Chinese adults is as high as 34% [15]. Previous studies revealed that several lipid-related genes are associated with different lipid traits in the Maonan and Han populations [16–18]. The Maonan ethnicity is one of 55 recognized minorities in China, and seventy percent (64,500) of Maonan people live in Huanjiang Maonan Autonomous County of Guangxi Zhuang Autonomous Region according to a wire report from Xinhua News Agency (Beijing, May 20th, 2020). The Maonan people maintain a unique lifestyle and wedding culture characterized by a fat-rich diet, recreational use of alcohol and tobacco, and intra-ethnic marriages. As a result, the Maonan population has a singular genetic background and specific environmental risk factors that are particularly informative in genetic studies of dyslipidemia. In this study, we therefore explored associations between *TUBB-WWOX* SNPs, their haplotypes, and gene-gene (G × G) and gene-environment (G × E) interactions and the prevalence of dyslipidemia in the Maonan population.

## RESULTS

### Participant characteristics

As shown in Table 1, no statistically significant differences in mean age, gender ratio, height, cigarette smoking, and proportion over 65 years old were observed between the normal and dyslipidemia groups (*P* > 0.05). However, mean weight, waist circumference, body mass index (BMI), alcohol consumption, systolic blood pressure (SBP), diastolic blood pressure (DBP), pulse pressure (PP), fasting blood-glucose (FBS), hypertension morbidity, and proportion with BMI greater than 24 kg/m^2^ and with FBS of at least 7.0 mM were higher in the dyslipidemia group than in the normal group (*P* < 0.01). In addition, total cholesterol (TC), triglyceride (TG), LDL-C, and apolipoprotein (Apo) B levels were also significantly higher in the dyslipidemia group than in the normal group (*P* < 0.001). In contrast, HDL-C and ApoA1 levels and ApoA1/ApoB ratio were significantly lower in the dyslipidemia group than in the normal group (*P* < 0.001).

**Table 1:** General and biochemical characteristics of the participants.

**Parameter**	**Normal**	**Dyslipidemia**	***t (χ^2^)***	***P***
Number	864	1129	–	–
Male/female	431/433	545/584	0.509	0.476
Age, years^1^	55.31±13.08	56.04±12.88	-1.240	0.215
Height, cm	156.34±8.01	156.75±8.32	-1.123	0.262
Weight, kg	54.48±110.29	57.06±11.07	-5.365	0.000
Waistline, cm	76.06±9.31	80.33±9.47	-8.199	0.000
Body mass index (BMI), kg/m^2^	22.21±3.31	23.11±3.39	-5.971	0.000
Cigarette smoking, n (%)^2^				
0 cigarette/day	622(71.99)	818(72.45)		
≤ 20 cigarettes/day	192(22.22)	239(21.17)		
> 20 cigarettes/day	50(5.79)	72(6.38)	0.544	0.762
Alcohol consumption, n (%)				
0 g/day	607(70.25)	841(74.49)		
≤ 25 g/day	50(5.79)	84(7.44)		
> 25 g/day	207(23.96)	204(18.07)	11.633	0.003
Systolic blood pressure, mmHg	127.80±20.45	135.34±22.30	-7.844	0.000
Diastolic blood pressure, mmHg	79.83±12.29	84.53±12.91	-8.276	0.000
Pulse pressure, mmHg	48.91±15.00	51.31±16.39	-3.404	0.001
Fasting blood-glucose (FBS), mM	6.03±0.94	6.32±1.23	-5.915	0.000
Total cholesterol, mM	4.33±0.50	4.89±0.72	-20.024	0.000
Triglyceride, mM	1.00(1.07)	1.56(2.02)	-28.603	0.000
HDL-C, mM	1.30±0.18	1.15±0.17	19.195	0.000
LDL-C, mM	2.90±0.15	3.43±0.31	-49.871	0.000
Apolipoprotein (Apo)A1, g/L	1.32±0.18	1.28±0.16	5.931	0.000
ApoB, g/L	0.94±0.14	1.00±0.10	-10.755	0.000
ApoA1/ApoB	1.45±0.32	1.29±0.24	11.697	0.000
Hypertension, n (%)	288(33.33)	571(43.96)	59.334	0.000
Age > 65 years	194(22.45)	255(22.59)	0.0050	0.944
BMI > 24 kg/m^2^	255(22.59)	475(42.07)	55.200	0.000
FBS ≥ 7.0 mM, n (%)	164(18.98)	495(43.84)	52.194	0.000

HDL-C, high-density lipoprotein cholesterol; LDL-C, low-density lipoprotein cholesterol. ^1^Normally distributed quantitative data were assessed in a *t*-test and are described as means ± SD. ^2^Qualitative data were assessed using the chi-square test. ^3^Non-normally distributed quantitative data were assessed using the Wilcoxon-Mann-Whitney test and are described as medians (interquartile range). *P* < 0.05 indicated a statistically significant difference.

### Genotype and allele frequencies

Information for four target SNPs (rs3132584, rs3130685, rs2222896, and rs2548861) in the *TUBB* and *WWOX* genes among the Chinese Han Beijing (CHB) population in NCBI dbSNP Build 132 is shown in Table 2; genotypic and allelic frequencies of these four SNPs in the Maonan population are presented in Table 3. The frequencies of point mutations at all four SNPs were similar between the CHB and Maonan populations. Mutations from G to T and from C to T were observed at the *TUBB* rs3132584 and rs3130685 SNPs, respectively, while mutations from A to G and from T to G were observed at the *WWOX* rs2222896 and rs2548861 SNPs, respectively. However, genotypic and allelic frequencies of the four SNPs differed between the ethnic groups; in particular, the frequency of the rs3132584TT genotype was 5.0% in Maonan population but was 0% in the CHB population. In this study, allelic and genotypic distributions of the four SNPs in both groups were consistent with the Hardy Weinberg Equilibrium (HWE, *P* > 0.05 for all). Additionally, allele and genotype frequencies of the rs3132584, rs3130685, and rs2222896 SNPs differed between the normal and dyslipidemia groups (*P* ≤ 0.01 for all).

**Table 2:** Frequencies of *TUBB* and *WWOX* SNPs in the Chinese Han Beijing population [*n*(%)].

**Gene**	**Chromosome**	**SNP**	**Allele_1_**	**Allele_2_**	***P*_HWE_**	**Genotype detail of CHB**			**Source**	**Phenotype**
*TUBB*	6	rs3132584	G(0.8171)	*T(0.1829)	0.4386	GG(0.6341)	GT(0.3591)	—	1000 Genome	↑LDL(G)
	6	rs3130685	*C(0.4556)	T(0.5444)	1.0000	CC(0.2000)	CT(0.5111)	TT(0.2889)	1000 Genome	↓LDL(T)
*WWOX*	16	rs2222896	*A(0.3659)	G(0.6341)	0.7518	AA(0.1220)	AG(0.3902)	GG(0.4878)	1000 Genome	↑LDL(G)
	16	rs2548861	*T(0.4512)	G(0.5488)	0.4028	TT(0.1707)	TG(0.5610)	GG(0.2683)	1000 Genome	↓HDL(T)

SNP, single nucleotide polymorphism; HWE, Hardy-Weinberg equilibrium; CHB, Chinese Han Beijing. Allele_1_ and the allele_2_ indicate reference and alternate alleles, respectively; *allele indicates the minor allele.

**Table 3:** Genotypic and allelic frequencies of *TUBB* and *WWOX* SNPs in normal and dyslipidemia groups [*n*(%)].

**Gene SNP**	**Genotype (allele)**	**Total (*n* =1993)**	**Normal (*n* = 864)**	**Dyslipidemia (*n* = 1229)**	***χ^2^***	***P***
*TUBB*						
rs3132854	GG	1223(61)	467(54)	756(67)		
	GT	676(34)	337(39)	339(30)		
	TT	94(5)	60(7)	34(3)	40.978	0.000
	G	3122(78)	1271(74)	1851(82)		
	T	864(22)	457(26)	407(18)	40.896	0.000
	*P*_HWE_	0.95	1.000	0.690		
rs3130685	CC	361(18)	138(16)	223(20)		
	CT	980(49)	415(48)	565(50)		
	TT	652(33)	311(36)	341(30)	9.282	0.010
	T	2284(57)	1037(60)	1247(55)		
	C	1702(43)	691(40)	1011(45)	9.163	0.002
	*P*_HWE_	0.850	1.000	0.720		
*WWOX*						
rs2222896	AA	395(20)	259(30)	136(12)		
	AG	961(48)	444(51)	517(46)		
	GG	637(32)	161(19)	476(42)	167.338	0.000
	G	2235(56)	766(44)	1469(65)		
	A	1751(44)	962(56)	789(35)	170.763	0.000
	*P*_HWE_	0.340	0.240	0.840		
rs2548861	TT	287(14)	118(14)	169(15)		
	TG	947(48)	404(47)	543(48)		
	GG	759(38)	342(40)	417(37)	1.670	0.434
	G	2465(62)	1088(63)	1377(61)		
	T	1521(38)	640(37)	881(39)	1.626	0.202
	*P*_HWE_	0.780	1.000	0.750		

Qualitative data were assessed using the Chi-square test; *P* < 0.05 indicated a statistically significant difference. HWE, Hardy-Weinberg equilibrium. *P*_HWE_ > 0.05 indicated a statistically significant difference.

### Associations between genotypes and alleles, serum lipid levels, and dyslipidemia

**Figure 1:**
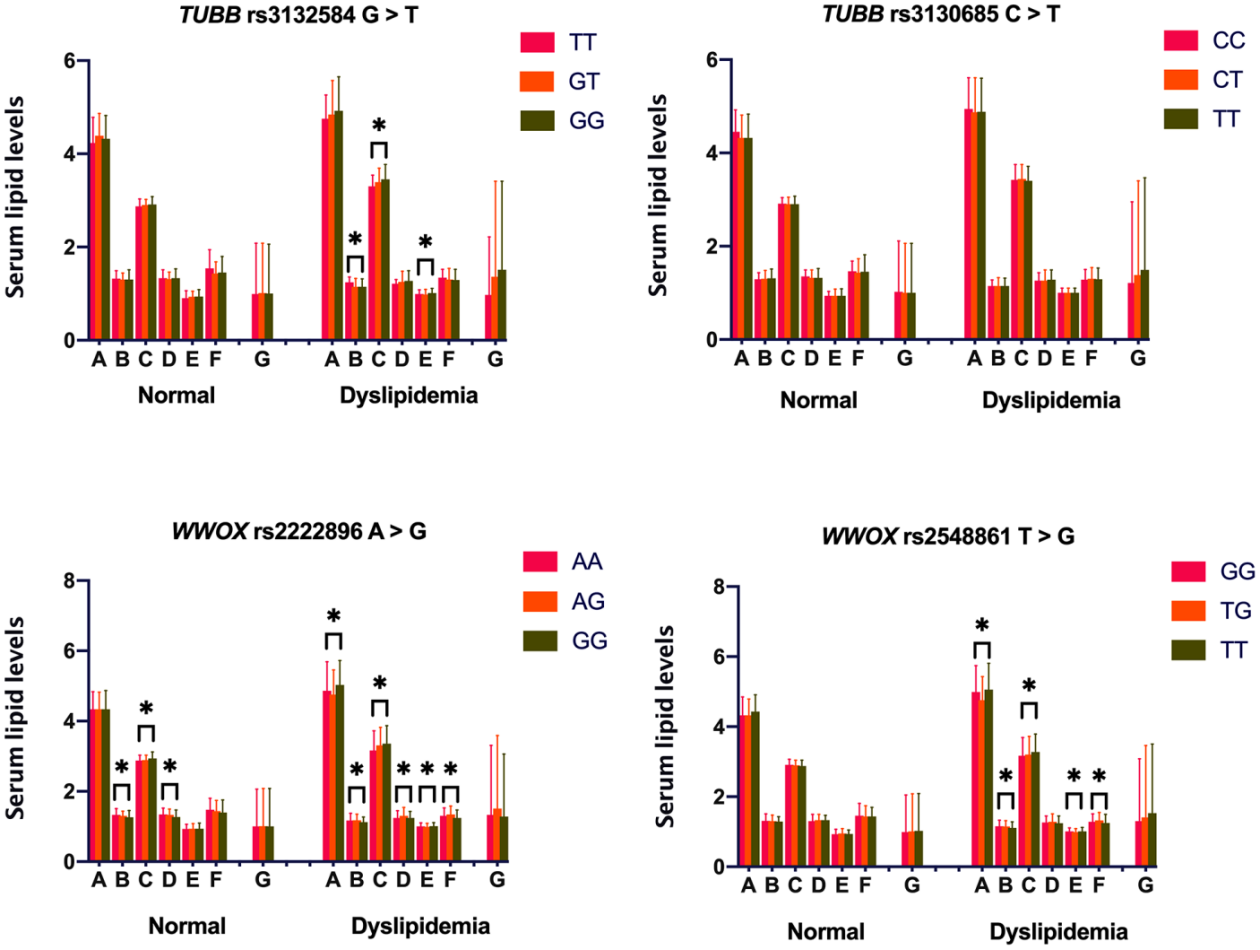
**Associations between *TUBB-WWOX* genotypes and serum lipid levels in normal and dyslipidemia groups.** A = Total cholesterol (mM); B = High-density lipoprotein cholesterol (mM); C = Low-density lipoprotein cholesterol (mM); D = Apolipoprotein (Apo) A1 (g/L); E = ApoB (g/L); F = ApoA1/ApoB ratio; and G = Triglyceride (mM). *P-value < 0.006 indicated a statistically significant difference after Bonferroni correction.

Associations between the four *TUBB* and *WWOX* SNPs and serum lipid levels are shown in Figure 1. Serum levels of HDL-C, LDL-C, and ApoA1 differed among the three rs2222896 genotypes in the normal group (*P* ≤ 0.001 for all); rs2222896G allele carriers had higher LDL-C and lower HDL-C levels than those without rs2222896G. However, there were no differences in serum lipid levels depending on genotype for the other three SNPs. In the dyslipidemia group, some lipid parameters differed significantly depending on genotype for the rs3132584, rs2222896, and rs2548861 SNPs (*P* < 0.0125 for all). Furthermore, higher TC levels and lower ApoA1/ApoB ratio were associated with rs2222896G and rs2548861T alleles, higher LDL-C and ApoB levels and lower HDL-C levels were associated with the rs3132584G, rs2222896G, and rs2548861T alleles, lower ApoA1 levels were associated with the rs2222896G allele, and lower ApoA1/ApoB ratio was associated with the rs2548861T and rs2222896G alleles.

Because environmental factors can affect lipid phenotypes, we adjusted for the risk factors of age, sex, drinking, smoking, FBS, and BMI, to clarify relationships between genotypes and lipid phenotypes (Table 4). Overall, in the normal group, serum HDL-C levels were lower in rs2222896G allele carriers than in rs2222896G non-carriers (*P* ≤ 0.001), LDL-C was higher, but ApoA1 was lower, in subjects with the rs2222896GG genotype compared to those with the rs2222896AA genotype (*P* < 0.001), ApoB levels were higher in subjects with the rs3132584GG genotype than in those with the rs3132584TT genotype (*P* = 0.006), and ApoA1/ApoB ratio was lower in subjects with the rs2222896GG and rs3132584GT genotypes than in those with the rs2222896AA and rs3132584TT genotypes, respectively (*P* = 0.012 and *P* = 0.007). In the dyslipidemia group, serum TC and ApoB levels were higher, but ApoA1/ApoB ratio was lower, in subjects with the rs2548861GG genotype than in those with the rs2548861TG genotype (*P* ≤ 0.005 for all), serum HDL-C and ApoA1 levels and ApoA1/ApoB ratio were lower, but LDL-C levels were higher, in subjects with the rs2222896GG genotype than in those with the rs2222896AA genotype, and serum HDL-C levels were lower in subjects with the rs3132584GT, rs3132584GG, and rs2548861TT genotypes than in those with the rs3132584TT and rs2548861GG genotypes (*P* = 0.001 for all).

**Table 4:** Meaningful associations between the four SNPs and serum lipid levels in the normal and dyslipidemia groups.

**Lipid**	**SNP**	**Genotype**		**Beta**	***t***	***P***	**95% CI of Beta**	
		**Reference**	**Alternate**				**Lower**	**Upper**
**Normal**								
HDL-C	rs2222896	AA	AG	-0.131	-3.313	0.001	-0.076	-0.019
	rs2222896	AA	GG	-0.165	-4.232	0.000	-0.112	-0.041
LDL-C	rs2222896	AA	GG	0.137	3.500	0.000	0.024	0.084
ApoA1	rs2222896	AA	GG	-0.160	-4.080	0.000	-0.109	-0.038
Apo B	rs3132584	TT	GG	0.188	2.739	0.006	0.015	0.090
ApoA1/ApoB	rs2222896	AA	GG	-0.099	-2.509	0.012	-0.143	-0.017
	rs3132584	TT	GT	-0.188	-2.708	0.007	-0.210	-0.034
**Dyslipidemia**								
TC	2548861	GG	TG	-0.156	-4.832	0.000	-0.318	-0.134
HDL-C	rs3132584	TT	GT	-0.272	-3.302	0.001	-0.159	-0.041
		TT	GG	-0.296	-3.598	0.000	-0.164	-0.048
	rs2222896	AA	GG	-0.155	-3.270	0.001	-0.085	-0.021
	2548861	GG	TT	-0.113	-3.471	0.001	-0.084	-0.023
LDL-C	rs2222896	AA	GG	0.136	2.843	0.005	0.027	0.146
ApoA1	rs2222896	AA	GG	-0.167	-3.535	0.000	-0.083	-0.024
Apo B	2548861	GG	TG	-0.089	-2.695	0.007	-0.031	-0.005
ApoA1/ApoB	rs2222896	AA	GG	-0.120	-2.549	0.011	-0.102	-0.013
	2548861	GG	TG	0.085	2.577	0.010	0.010	0.070

TC, total cholesterol; HDL-C, high-density lipoprotein cholesterol; LDL-C, low-density lipoprotein cholesterol; ApoA1, apolipoprotein A1; ApoB, apolipoprotein B; CI, confidence interval. *P* values were calculated by multivariable linear regression. *P* < 0.0125 indicated a statistically significant difference after Bonferroni correction and adjusting for genotype, age, gender, drinking, smoking, fasting blood-glucose, and body mass index.

Associations between the four SNPs and dyslipidemia are shown in Table 5. rs3132584, rs3130685, and rs2222896 were associated with dyslipidemia (*P* < 0.05). Subjects with the rs3132584TT [codominant: adjusted odds ratio (OR) = 0.35, 95% confidence interval (CI) = 0.23–0.54, *P* < 0.0001] or rs3132584GT+TT (dominant: adjusted OR = 0.58, 95% CI = 0.48–0.70, *P* < 0.0001) genotypes had a lower risk of dyslipidemia than those with the rs3132584GG genotype. In contrast, subjects with the rs3130685CC (codominant: adjusted OR = 1.47, 95% CI = 1.13–1.92, *P* = 0.0096) or rs3130685 CT+TT (dominant: adjusted OR = 1.30, 95% CI = 1.08–1.57, *P* = 0.0064) genotypes had a higher risk of dyslipidemia than those with the rs3130685TT genotype. Finally, subjects with the rs2222896AA (codominant: adjusted OR = 0.18, 95% CI = 0.14–0.23, *P* < 0.0001) or rs2222896 AG+AA (dominant: adjusted OR = 0.31, 95% CI = 0.26–0.39, *P* < 0.0001) genotypes had a lower risk of dyslipidemia than those with the rs2222896GG genotype.

**Table 5:** Associations between genetic models of the four SNPs and dyslipidemia.

**Gene**	**Model**	**Genotype**		***χ^2^***	***P***	**OR (95% CI)**	*P
		**Reference**	**Alternate**				
***TUBB***							
rs3132584 G > T	Codominant	GG	GT	24.275	0.000	0.62(0.51–0.75)	< 0.0001
			TT	23.917	0.000	0.35(0.23–0.54)	
	Dominant	GG	GT+TT	34.413	0.000	0.58(0.48–0.70)	< 0.0001
	Recessive	GG+GT	TT	16.846	0.000	0.42(0.27–0.64)	< 0.0001
	Log-additive	GT	TT	6.456	0.011	0.61(0.52–0.71)	< 0.0001
rs3130685C> T	Codominant	TT	CT	4.542	0.033	1.24(1.02–1.52)	0.0096
			CC	8.448	0.004	1.47(1.13–1.92)	
	Dominant	TT	CT+CC	7.458	0.006	1.30(1.08–1.57)	0.0064
	Recessive	TT+CT	CC	4.714	0.030	1.29(1.03–1.64)	0.029
	Log-additive	CT	CC	1.848	0.174	1.22(1.07–1.38)	0.0024
***WWOX***							
rs2222896A>G	Codominant	GG	AG	71.311	0.000	0.39(0.32–0.49)	< 0.0001
			AA	164.027	0.000	0.18(0.14–0.23)	
	Dominant	GG	AG+AA	124.580	0.000	0.31(0.26–0.39)	< 0.0001
	Recessive	GG+AG	AA	99.024	0.000	0.32(0.25–0.40)	< 0.0001
	Log-additive	AG	AA	42.060	0.000	0.42(0.37–0.48)	< 0.0001
rs2548861T>G	Codominant	GG	TG	0.985	0.321	1.10(0.91–1.34)	0.4300
			TT	1.315	0.251	1.17(0.89–1.55)
	Dominant	GG	TG+TT	1.455	0.228	1.12(0.93–1.34)	0.2300
	Recessive	GG+TG	TT	0.683	0.409	1.11(0.86–1.43)	0.4100
	Log-additive	TG	TT	0.216	0.642	1.09(0.96–1.24)	0.2000

OR, odds ratio; CI, confidence interval. *P* and *P < 0.0125 indicated a statistically significant difference.

### Associations between haplotype frequencies, serum lipid levels, and dyslipidemia

**Figure 2:**
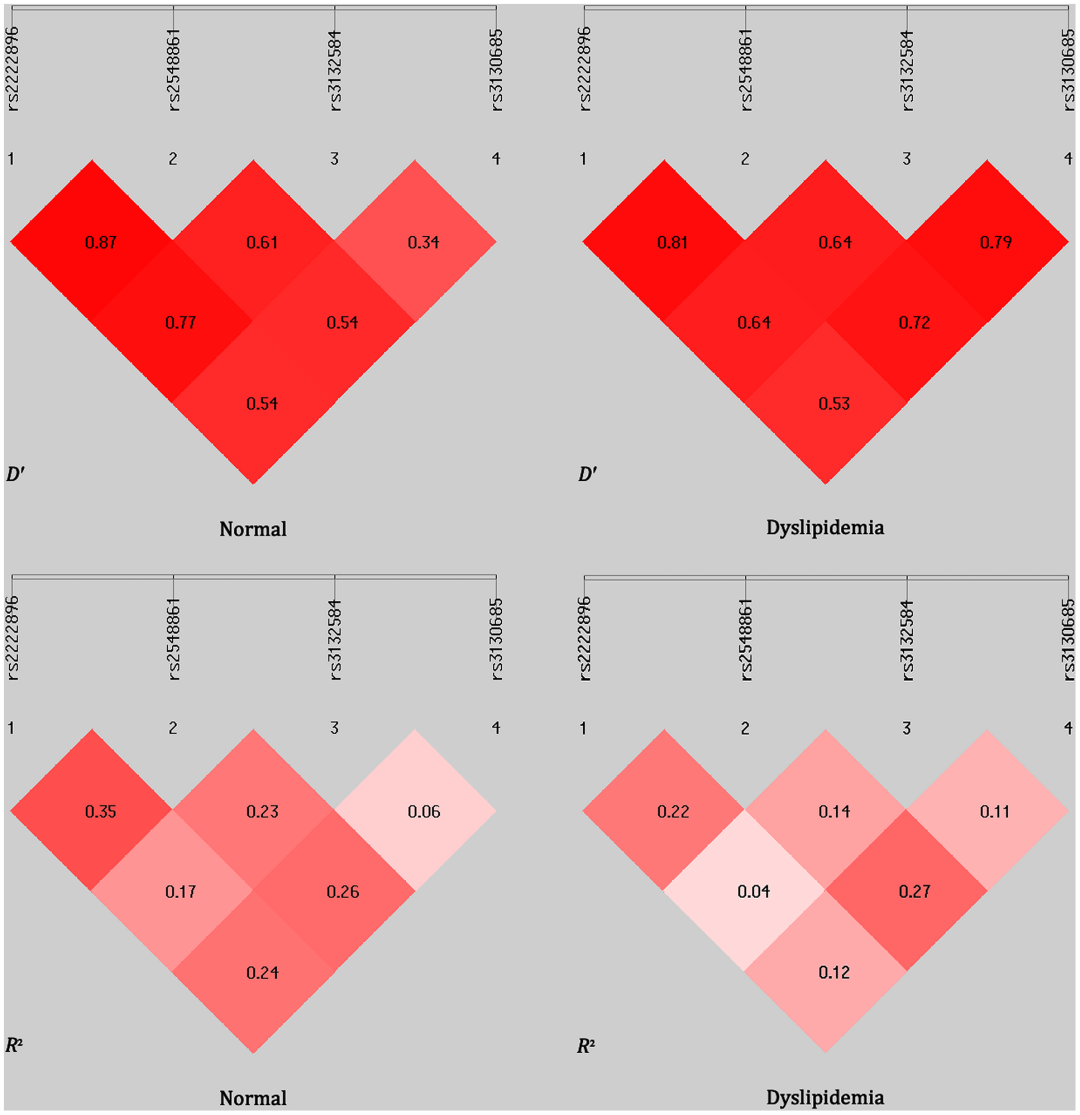
**Linkage disequilibrium (LD) analysis for the four *TUBB* and *WWOX* SNPs in normal and dyslipidemia groups.** LD status is indicated by *D*′ and *R*^2^ values.

Linkage disequilibrium (LD) was observed between the *WWOX* rs2222896 and rs2548861 SNPs in the normal and dyslipidemia groups (*D*′ = 0.870 or *R*^2^ = 0.350 and *D*′ = 0.810 or *R*^2^ = 0.220, respectively; Figure 2). Frequencies of the common haplotypes [lowest frequency threshold (LFT) > 0.03] of the two *WWOX* SNPs are shown in Table 6. The frequencies of the rs2222896A-rs2548861T, rs2222896G-rs2548861G, rs2222896A-rs2548861G, and rs2222896G-rs2548861T haplotypes differed between the two groups (*P* < 0.05 for all). The rs2222896G-rs2548861G and rs2222896G-rs2548861T haplotypes were associated with increased risk of dyslipidemia, whereas the rs2222896A-rs2548861T and rs2222896A-rs2548861G haplotypes were protective factors against dyslipidemia (*P* < 0.007 for all).

**Table 6:** Haplotype frequencies of the two *WWOX* SNPs in the normal and dyslipidemia groups [*n*(frequency)].

**Label**	**Haplotype**	**Normal (*n*=1728)**	**Dyslipidemia (*n*=2258)**	***χ^2^***	***P***	**OR (95% CI)**	*P
H1	rs2222896A–rs2548861T	604(0.349)	451(0.199)	112.869	2.30× 10^−26^	0.464(0.420–0.535)	< 0.0001
H2	rs2222896G–rs2548861G	730(0.422)	1039(0.460)	5.632	0.017	1.165(1.026–1.322)	0.003
H3	rs2222896A–rs2548861G	358(0.207)	338(0.149)	22.445	2.16× 10^−6^	0.673(0.571–0.793)	0.007
H4	rs2222896G–rs2548861T	36(0.020)	430(0.190)	272.729	2.88× 10^−61^	11.055(7.818–15.633)	< 0.0001

H, haplotype; OR, odds ratio; CI, confidence interval. *P* and *P < 0.05 indicated a statistically significant difference. *n* = total number with that haplotype.

**Figure 3:**
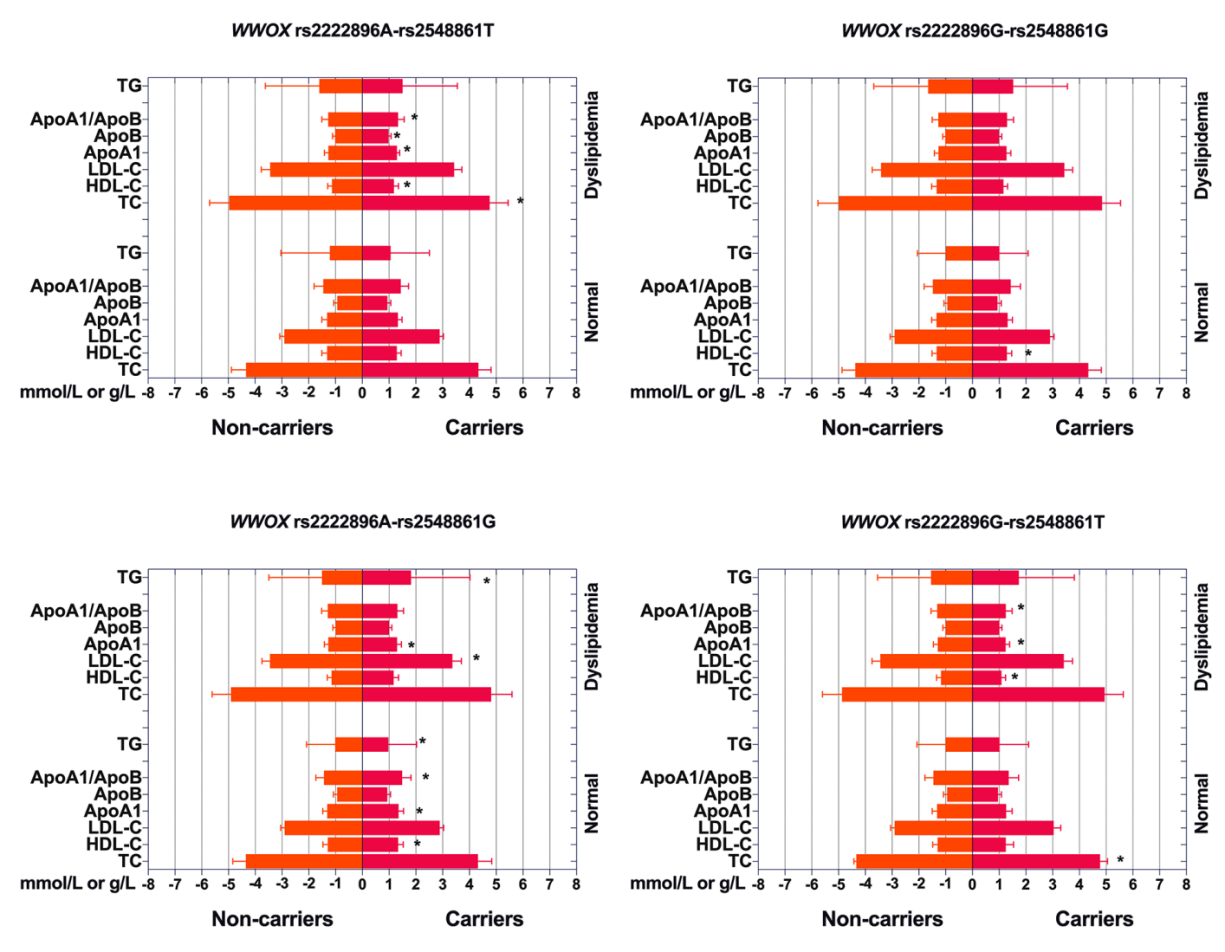
**Associations between *WWOX* haplotypes, serum lipid levels, and dyslipidemia.** *P-value < 0.006 indicated a statistically significant difference after Bonferroni correction.

Associations between the four haplotypes and serum lipid levels are shown in Figure 3. rs2222896G-rs2548861G haplotype carriers in the normal group had lower HDL-C levels than those without rs2222896G-rs2548861G haplotype (*P* = 0.001), rs2222896A-rs2548861G haplotype carriers had higher ApoA1 and HDL-C levels and ApoA1/ApoB ratio, but lower TG levels, than those without rs2222896A-rs2548861G haplotype (*P* ≤ 0.002), and rs2222896G-rs2548861T haplotype carriers had higher TC levels than those without rs2222896G-rs2548861T haplotypes (*P* < 0.001). In the dyslipidemia group, rs2222896A-rs2548861T haplotype carriers had lower TC and ApoB levels, but higher HDL-C and ApoA levels and ApoA1/ApoB ratio than those without corresponding haplotype (*P* < 0.001), rs2222896A-rs2548861G haplotype carriers had lower LDL-C and higher ApoA1 and TG levels than those without corresponding haplotype (*P* ≤ 0.004), and rs2222896G-rs2548861T haplotype carriers had lower ApoA1 and HDL-C levels and ApoA1/ApoB ratio than those without corresponding haplotype (*P* < 0.001).

As shown in Table 7, after adjusting for sex, age, BMI, FBS, smoking, and drinking in the normal group, the rs2222896G-rs2548861T haplotype was associated with increased levels of serum TC and LDL-C (*P* < 0.001), the rs2222896A-rs2548861G haplotype was associated with increased serum HDL-C and ApoA1 levels and ApoA1/ApoB ratio, but decreased serum TG levels (*P* ≤ 0.006), and the rs2222896G-rs2548861G haplotype was associated with decreased serum HDL-C levels (*P* < 0.001). In the dyslipidemia group, the rs2222896A-rs2548861T haplotype was associated with decreased serum TC and ApoB levels and increased serum HDL-C and ApoA1 levels and ApoA1/ApoB ratio (*P* < 0.001), the rs2222896A-rs2548861G haplotype was associated with decreased serum LDL-C levels and increased serum ApoA1 (*P* ≤ 0.006) and TG levels (*P* < 0.001), and the rs2222896G-rs2548861T haplotype was associated with decreased serum ApoA1 and HDL-C levels and ApoA1/ApoB ratio (*P* < 0.001).

**Table 7:** Association between *WWOX* haplotypes and serum lipid levels in normal and dyslipidemia groups.

**Lipid**	**Haplotype**	**Subjects**	**Beta**	***t***	***P***	**95%CI of Beta**	
						**Lower**	**Upper**
**Normal**							
TC	rs2222896G–rs2548861T	carriers/non-carriers	0.128	3.765	0.000	0.212	0.673
TG	rs2222896A–rs2548861G	carriers/non-carriers	-0.167	-4.912	0.000	-0.085	-0.036
HDL-C	rs2222896A–rs2548861G	carriers/non-carriers	0.151	4.414	0.000	0.034	0.089
	rs2222896G–rs2548861G	carriers/non-carriers	-0.124	-3.610	0.000	-0.074	-0.022
LDL-C	rs2222896G–rs2548861T	carriers/non-carriers	0.136	4.012	0.000	0.074	0.217
ApoA1	rs2222896A–rs2548861G	carriers/non-carriers	0.109	3.163	0.002	0.017	0.071
ApoA1/ApoB	rs2222896A–rs2548861G	carriers/non-carriers	0.095	2.772	0.006	0.020	0.116
**Dyslipidemia**							
TC	rs2222896A–rs2548861T	carriers/non-carriers	-0.138	-4.632	0.000	-0.290	-0.118
	rs2222896G–rs2548861G	carriers/non-carriers	-0.083	-2.796	0.005	-0.233	-0.041
TG	rs2222896A–rs2548861G	carriers/non-carriers	0.126	4.207	0.000	0.093	0.256
HDL-C	rs2222896A–rs2548861T	carriers/non-carriers	0.136	4.530	0.000	0.026	0.067
	rs2222896G–rs2548861T	carriers/non-carriers	-0.193	-6.556	0.000	-0.099	-0.053
LDL-C	rs2222896A–rs2548861G	carriers/non-carriers	-0.120	-4.006	0.000	-0.135	-0.046
ApoA1	rs2222896A–rs2548861T	carriers/non-carriers	0.121	4.053	0.000	0.020	0.058
	rs2222896A–rs2548861G	carriers/non-carriers	0.083	2.773	0.006	0.009	0.054
	rs2222896G–rs2548861T	carriers/non-carriers	-0.117	-3.931	0.000	-0.064	-0.022
ApoB	rs2222896A–rs2548861T	carriers/non-carriers	-0.117	-3.913	0.000	-0.036	-0.012
ApoA1/ApoB	rs2222896A–rs2548861T	carriers/non-carriers	0.142	4.769	0.000	0.040	0.097
	rs2222896G–rs2548861T	carriers/non-carriers	-0.106	-3.570	0.000	-0.091	-0.026

TC, total cholesterol; TG, triglyceride; HDL-C, high-density lipoprotein cholesterol; LDL-C, low-density lipoprotein cholesterol; ApoA1, apolipoprotein A1; ApoB, apolipoprotein B; CI, confidence interval. *P* values were calculated by multivariable linear regression. *P* < 0.0125 indicated a statistically significant difference after Bonferroni correction and adjusting for genotype, age, gender, drinking, smoking, fasting blood-glucose, and body mass index.

**Figure 4:**
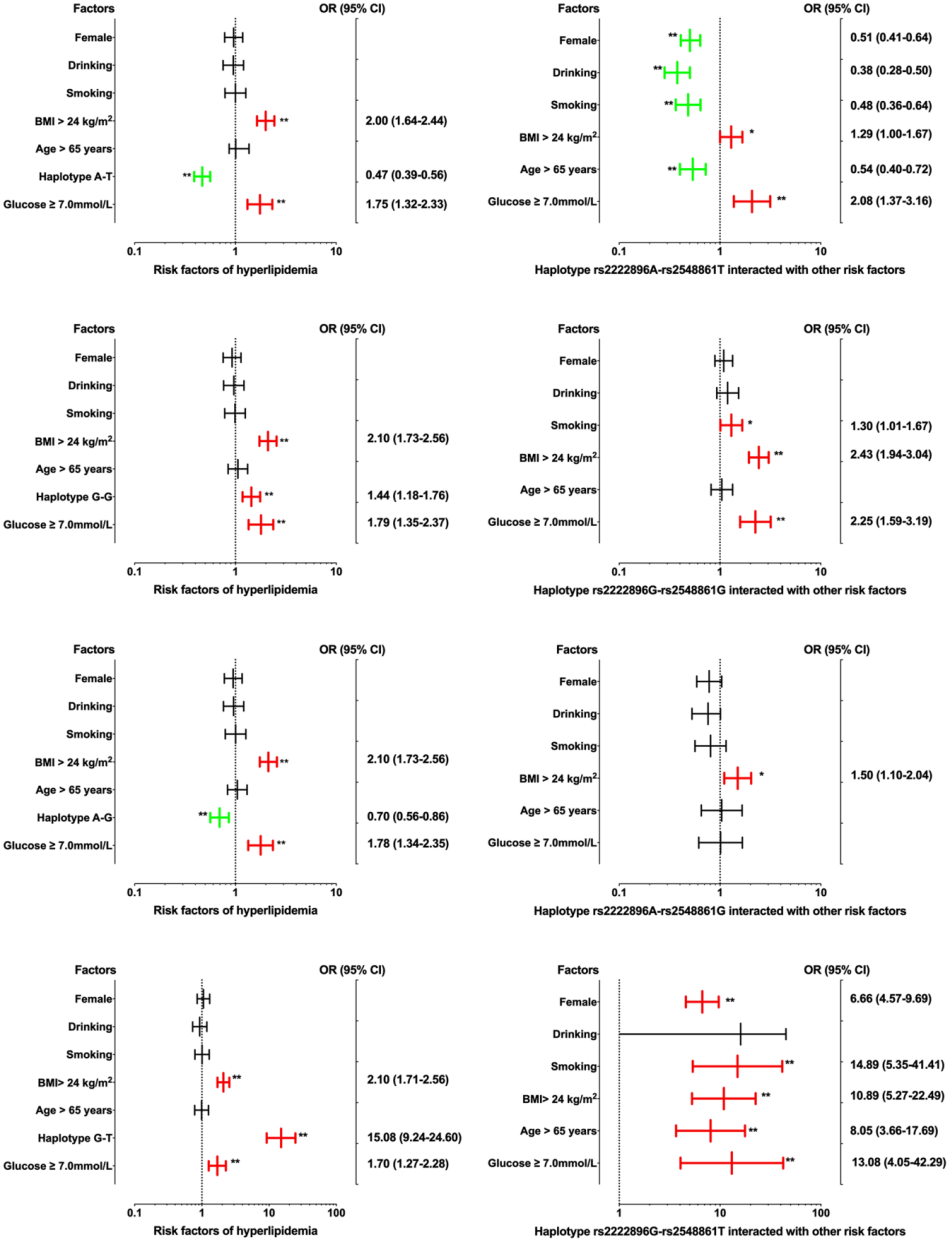
**Associations between *WWOX* haplotypes, environmental factors, and their interactions on dyslipidemia.** *P-value < 0.05 and **P-value < 0.001.

Interactions between the four haplotypes and environmental factors on dyslipidemia are shown in Figure 4. Dyslipidemia morbidity was increased in those with the rs2222896G-rs2548861G and rs2222896G-rs2548861T haplotypes compared to those without corresponding haplotypes (OR = 1.44, 95% CI = 1.18−1.76, *P* < 0.001 and OR = 15.08, 95% CI = 9.24−24.60, *P* < 0.001, respectively); environmental factors such as BMI > 24 kg/m^2^ and FBS ≥ 7.0 mM also increased dyslipidemia morbidity. In contrast, dyslipidemia morbidity was decreased in those with the rs2222896A-rs2548861T and rs2222896A-rs2548861G haplotypes compared to those without corresponding haplotypes (OR = 0.47, 95% CI = 0.39−0.56, *P* < 0.001 and OR = 0.70, 95% CI = 0.56−0.86, *P* < 0.001, respectively). Interactions between haplotype and environmental factors had different effects on the incidence of dyslipidemia than those observed for each individual locus. Interactions between the rs2222896G-rs2548861G and rs2222896G-rs2548861T haplotypes and smoking, high BMI, or FBS increased the risk of dyslipidemia, as did the interaction between the rs2222896G-rs2548861T haplotype and sex or age (*P* < 0.05 for all). Furthermore, interactions between the rs2222896A-rs2548861T haplotype and sex, age, BMI, drinking, or smoking, and between the rs2222896G-rs2548861T haplotype and BMI or FBS decreased the effects of the environmental factors on dyslipidemia (*P* < 0.05 for all).

### Associations between G × G interactions and dyslipidemia

As shown in Table 8, thirteen *TUBB-WWOX* G **×** G interactions were observed in the normal and dyslipidemia groups (LEF for gene interactions > 0.03). Frequencies of nine of these G × G interactions, namely G-C-A-T, G-C-A-G, G-C-G-T, T-T-A-G, T-T-G-T, T-T-G-G, T-C-G-G, T-T-A-T, and T-C-G-T, differed significantly between the two groups (*P* < 0.05). Among them, only the T-T-G-T interaction was associated with increased incidence of dyslipidemia (OR = 1.371, 95% CI = 1.112–1.689, *P* = 0.003), while the other eight interactions, particularly T-T-G-G, were associated with reduced incidence of dyslipidemia (OR = 0.795, 95% CI = 0.640–0.988, *P* = 0.038).

**Table 8:** Frequencies of gene-gene interactions in the normal and dyslipidemia groups [*n* (frequency)].

**Label**	**Gene-gene interaction**				**Normal**		**Dyslipidemia**		***χ^2^***	***P***	**OR (95% CI)**
	G1	G2	G3	G4							
I 1	G	C	A	T	412	(0.238)	260	(0.115)	128.782	0.000	0.328(0.217–0.398)
I 2	G	C	A	G	489	(0.283)	449	(0.199)	55.068	0.000	0.506(0.423–0.606)
I 3	G	C	G	T	375	(0.217)	388	(0.172)	16.853	0.000	0.683(0.569–0.819)
I 4	G	C	G	G	479	(0.277)	612	(0.271)	0.300	0.584	0.951(0.796–1.137)
I 5	G	T	A	T	357	(0.207)	388	(0.172)	1.800	0.180	0.884(0.373–1.059)
I 6	G	T	G	G	523	(0.303)	634	(0.281)	3.847	0.050	0.835(0.697–1.000)
I 7	T	T	A	G	185	(0.107)	196	(0.087)	5.180	0.023	0.771(0.616–0.965)
I 8	T	T	G	T	185	(0.107)	307	(0.136)	8.761	0.003	1.371(1.112–1.689)
I 9	T	T	G	G	199	(0.115)	217	(0.096)	4.926	0.038	0.795(0.640–0.988)
I 10	G	T	A	G	470	(0.272)	576	(0.255)	2.241	0.134	0.873(0.731–1.043)
I 11	T	C	G	G	257	(0.149)	108	(0.048)	121.925	0.000	0.250(0.195–0.320)
I 12	T	T	A	T	234	(0.135)	180	(0.080)	36.219	0.000	0.511(0.410–0.636)
I 13	T	C	G	T	211	(0.122)	102	(0.045)	81.633	0.000	0.307(0.238–0.397)

G1, *TUBB* rs3132584 G>T; G2, *TUBB* rs3130685 C>T; G3, *WWOX* rs2222896 A>G; G4, *WWOX* rs2548861 T>G; I, interaction. Gene interaction frequencies < 0.03 in the two groups were excluded.

### Models of the effects of different interactions on dyslipidemia

Using generalized multifactor dimensionality reduction (GMDR) analysis and adjusting by covariates, we identified the fourteen best interactive models of SNP-SNP, haplotype-haplotype, G × G, and G × E effects on dyslipidemia (Table 9). The two- to four-locus interactive models for SNP-SNP and SNP-environment were rs2222896-rs3130685, rs2222896-rs3130685-rs2548861, rs2222896-rs3130685-rs2548861-rs3132584, rs2222896-rs3130685-age > 65 years, rs2222896-rs3132584-FBS ≥ 7.0 mM, and rs2222896-rs3132584-drinking (cross-validation (CV) constancy of 10 of 10, balanced accuracy test (Bal. Acc.) ≥ 64.98%, and permutation test *P* < 0.001 for all). The two- to three-locus interactive models for haplotype-haplotype and haplotype-environment were H1-H3 (interaction of rs2222896A-rs2548861T and rs2222896A-rs2548861G haplotypes), H2-H3-H4 (interaction of rs2222896G-rs2548861G, rs2222896A-rs2548861G, and rs2222896G-rs2548861T haplotypes), H1-H3-BMI > 24 kg/m^2^, and H1-H3-FBS ≥ 7.0 mM (CV constancy of 10 of 10, Bal. Acc. ≥ 62.05%, and permutation test *P* < 0.001 for all). Additionally, the two- to three-locus models of G × G and G × E interactions were I1-I2 (G-C-A-T and G-C-A-G), I3-I12 (G-C-G-T and T-T-A-T), I4-I8-I11 (G-C-G-G, T-T-G-T, and T-C-G-G), I4-I5-I10 (G-C-G-G, G-T-A-T, and G-T-A-G), and I1-I11-BMI > 24 kg/m^2^ (CV constancy of 10 of 10, Bal. Acc. ≥ 56.87%, and permutation test *P* < 0.001 for all).

**Table 9:** Best models for different types of interactions.

**Interactive model**	**Training Bal. Acc.**	**Testing Bal. Acc.**	**CV consistency**	**Sign test *P***	**Permutation test *P***
**SNP-SNP**					
A-B	0.7189	0.7208	10/10	0.001	<0.0001
A-B-C	0.8061	0.8043	10/10	0.001	<0.0001
A-B-C-D	0.8434	0.8290	10/10	0.001	<0.0001
**SNP-environment**					
A-B-F	0.7189	0.7208	10/10	0.001	<0.0001
A-D-H	0.6492	0.6509	10/10	0.001	<0.0001
A-D-J	0.6483	0.6498	10/10	0.001	<0.0001
**Haplotype–haplotype**					
H1-H3	0.6176	0.6176	10/10	<0.0001	<0.0001
**Haplotype-environment**					
H1-H3-G	0.6469	0.6469	10/10	<0.0001	<0.0001
H1-H3-H	0.6381	0.6369	10/10	<0.0001	<0.0001
**Gene-gene**					
I1-I2	0.6233	0.6233	10/10	<0.0001	<0.0001
I3-I12	0.5687	0.5687	10/10	<0.0001	<0.0001
I4-I8-I11	0.6950	0.6950	10/10	<0.0001	<0.0001
I4-I5-I10	0.6488	0.6488	10/10	<0.0001	<0.0001
**Gene-environment**					
I1-I11-G	0.6302	0.6268	10/10	<0.0001	<0.0001

A = rs2222896, B = rs3130685, C = rs2548861, D = rs3132584, E = female, F = age > 65 years, G = body mass index > 24 kg/m^2^, H = fasting blood-glucose ≥ 7.0 mM, I = smoking, J = drinking; Bal. Acc., balanced accuracy; CV, cross-validation.

**Figure 5:**
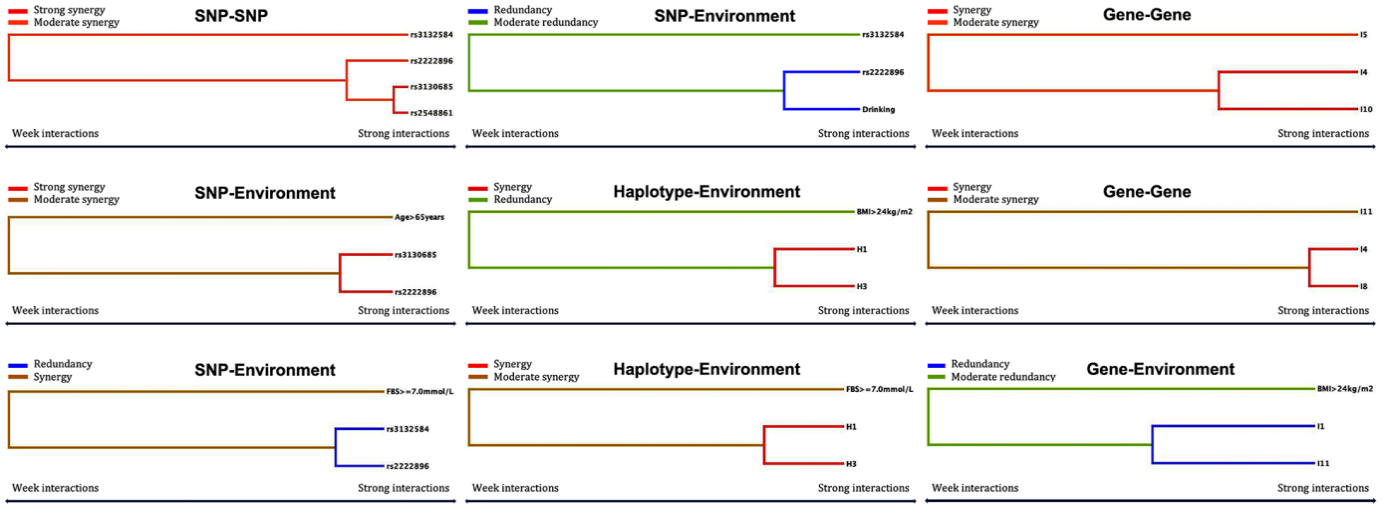
**Optimal interactions affecting dyslipidemia shown in a dendrogram.** Elements that interact strongly with each other appear close together in the leaves of the tree, while elements that interact weakly appear far from each other.

Furthermore, associations between the fourteen interactions and dyslipidemia were further verified through logistic regression analysis as shown in Figure 5 and Table 10. The following interactions between genotypes, haplotypes, and factors increased the risk of dyslipidemia: rs3130685TT-rs2548861TG+TT (OR = 3.099, 95% CI = 2.397–4.005, *P* < 0.001) and rs3130685CT+CC-rs2548861GG (OR = 3.136, 95% CI = 2.489–3.952, *P* < 0.001) compared to rs3130685TT-rs2548861GG; rs3130685TT-rs2222896AG+GG (OR = 2.532, 95% CI = 1.982–3.263, *P* < 0.001) and rs3132584GT+GG-rs2222896AG+GG (OR = 2.548, 95% CI = 2.035–3.189, *P* < 0.001) compared to rs3130685TT-rs2222896AA; rs2222896AG+GG-nondrinking compared to rs2222896AA-nondrinking (OR = 1.792, 95% CI = 1.490–2.155, *P* < 0.001); H1-H3 compared to H1–H3 non-carriers (OR = 3.315, 95% CI = 2.681–4.098, *P* < 0.001); I4 non-carriers-I10 carriers (OR = 2.295, 95% CI = 1.783–2.954, *P* < 0.001) and I4 carriers-I10 non-carriers (OR = 2.877, 95% CI = 2.253–3.675, *P* < 0.001) compared to I4–I10 non-carriers; and I4 non-carriers-I8 carriers (OR = 5.292, 95% CI = 3.694–7.582, *P* < 0.001) and I4 carriers-I8 non-carriers (OR = 1.465, 95% CI = 1.219–1.760, *P* < 0.001) compared to I4–I8 non-carriers. In contrast, interactions between the following genotypes, phenotypes, and factors decreased the risk of dyslipidemia: rs3132584GT+GG-rs2222896AA compared to rs3132584TT-rs2222896AA (OR = 0.441, 95% CI = 0.345–0.563, *P* < 0.001); rs2222896AA-drinking compared to rs2222896AA-nondrinking (OR = 0.298, 95% CI = 0.196–0.455, *P* < 0.001); and I1-I1 carriers compared to I1-I11 non-carriers (OR = 0.114, 95% CI = 0.097–0.164, *P* < 0.001).

**Table 10:** Risk of dyslipidemia with different types of interactions.

**Variable 1**	**Variable 2**	**OR (95% CI)**	***P***
**SNP-SNP interaction**			
rs3130685	rs2548861		
TT	GG	1	–
TT	TG+TT	3.099(2.397–4.005)	0.000
CT+CC	GG	3.136(2.489–3.952)	0.000
CT+CC	TG+TT	0.588(0.489–0.706)	0.000
rs3130685	rs2222896		
TT	AA	1	–
TT	AG+GG	2.532(1.982–3.236)	0.000
CT+CC	AA	1.317(0.929–1.867)	0.123
CT+CC	AG+GG	1.236(1.027–1.486)	0.025
rs3132584	rs2222896		
TT	AA	1	–
TT	AG+GG	1.526×10^9^(0.000–NA)	0.998
GT+GG	AA	0.441(0.345–0.563)	0.000
GT+GG	AG+GG	2.548(2.035–3.189)	0.000
**SNP-environment interaction**			
rs2222896	Drinking		
AA	No	1	–
AA	Yes	0.298(0.196–0.455)	0.000
AG+GG	No	1.792(1.490–2.155)	0.000
AG+GG	Yes	1.344(1.077–1.678)	0.009
**Haplotype-haplotype interaction**			
H1	H3		
Non-carriers	Non–carriers	3.315(2.681–4.098)	0.000
Non-carriers	Carriers	0.704(0.563–0.881)	0.002
Carriers	Non–carriers	0.485(0.403–0.583)	0.000
Carriers	Carriers	1	–
**Gene-gene interaction**			
I1	I11		
Non-carriers	Non-carriers	1	–
Non-carriers	Carriers	1.271(0.862–1.875)	0.226
Carriers	Non-carriers	0.808(0.648–1.006)	0.057
Carriers	Carriers	0.114(0.079–0.164)	0.000
I4	I8		
Non-carriers	Non-carriers	1	–
Non-carriers	Carriers	5.292(3.694–7.582)	0.000
Carriers	Non-carriers	1.465(1.219–1.760)	0.000
Carriers	Carriers	0.394(0.294–0.529)	0.000
I4	I10		
Non-carriers	Non-carriers	1	–
Non-carriers	Carriers	2.295(1.783–2.954)	0.000
Carriers	Non-carriers	2.877(2.253–3.675)	0.000
Carriers	Carriers	0.483(0.399–0.586)	0.000

H1, rs2222896A-rs2548861T haplotype; H3, rs2222896A-rs2548861G haplotype; I1, rs3132584G-rs3130685C-rs2222896A-rs2548861T; I4, rs3132584G-rs3130685C-rs2222896G-rs2548861G; I8, rs3132584T-rs3130685T-rs2222896G-rs2548861T; I10, rs3132584G-rs3130685T-rs2222896A-rs2548861G; I11, rs3132584T-rs3130685C-rs2222896G-rs2548861G; OR, odds ratio; CI, confidence interval. Different types of interactions were analyzed by logistic regression. *P* < 0.006 indicated a statistically significant difference after Bonferroni correction and adjusting for body mass index and fasting blood-glucose.

### Risk of hypertension based on different interactive models of dyslipidemia

The effects of traditional and potential genetic risks associated with dyslipidemia on hypertension are shown in Tables 11, 12. In this study, dyslipidemia, female sex, age > 65 years, BMI > 24 kg/m^2^, and FBS ≥ 7.0 mM were clear risk factors for hypertension (*P* < 0.05). Moreover, interactions between the rs3132584 and rs2222896 SNPs increased the risk of hypertension 1.523-fold (*P* < 0.006) after adjustment for BMI, sex, age, and FBS.

**Table 11:** Risk of hypertension in the study population [*n*(frequency)].

**Risk factor**	**Normal (*n*)**	**Hypertension (*n*)**	***χ^2^***	***P***	**OR (95% CI)**
Female	1017(0.510)	976(0.490)	3.936	0.047	1.235(1.003–1.522)
Smoking	1442(0.724)	551(0.276)	0.133	0.715	0.956(0.751–1.217)
Drinking	1448(0.727)	545(0.273)	1.303	0.254	1.149(0.905–1.457)
Dyslipidemia	864(0.434)	1129(0.566)	42.037	0.000	1.884(1.556–2.282)
Age > 65 years	1544(0.775)	449(0.225)	55.483	0.000	2.369(1.888–2.927)
BMI > 24 kg/m^2^	1293(0.649)	700(0.351)	25.641	0.000	1.660(1.364–2.020)
FBS ≥ 7.0 mM	1725(0.866)	268(0.134)	29.408	0.000	2.139(1.625–2.816)

BMI, body mass index; FBS, fasting blood-glucose; OR, odds ratio; CI, confidence interval. *P* < 0.05 indicated a statistically significant difference.

**Table 12:** Effects of meaningful interactive models of dyslipidemia on hypertension.

**Variable 1**	**Variable 2**	**OR (95% CI)**	***P***
**SNP-SNP interaction**			
rs3130685	rs2548861		
TT	GG	1	–
CT+CC	TG+TT	0.936(0.771–1.137)	0.506
rs3130685	rs2222896		
TT	AA	1	–
CT+CC	AG+GG	0.895(0.739–1.085)	0.259
rs3132584	rs2222896		
TT	AA	1	–
GT+GG	AG+GG	1.523(1.189–1.950)	0.001
**SNP-environment interaction**			
rs2222896	Drinking		
AA	No	1	–
AG+GG	Yes	1.264(0.997–1.603)	0.053
**Haplotype-haplotype interaction**			
H1	H3		
Non-carriers	Non-carriers	0.917(0.525–1.601)	0.761
Carriers	Carriers	1	–
**Gene-gene interaction**			
I1	I11		
Non-carriers	Non-carriers	1	–
Carriers	Carriers	0.448(0.202–0.993)	0.048
I4	I8		
Non-carriers	Non-carriers	1	–
Carriers	Carriers	0.819(0.417–1.609)	0.562
I4	I10		
Non-carriers	Non-carriers	1	–
Carriers	Carriers	0.650(0.411–1.026)	0.064

H1, rs2222896A-rs2548861T haplotype; H3, rs2222896A-rs2548861G haplotype; I1, rs3132584G-rs3130685C-rs2222896A-rs2548861T; I4, rs3132584G-rs3130685C-rs2222896Grs2548861G; I8, rs3132584T-rs3130685T-rs2222896G-rs2548861T; I10, rs3132584G-rs3130685T-rs2222896A-rs2548861G; I11, rs3132584T-rs3130685C-rs2222896G-rs2548861G; OR, odds ratio; CI, confidence interval. Different types of interactions were analyzed by logistic regression. *P* < 0.006 indicated a statistically significant difference after Bonferroni correction and adjusting for body mass index and fasting blood-glucose.

## DISCUSSION

Dyslipidemia, a multifactorial non-communicable chronic disease, contributes to pathogenesis of arteriosclerotic diseases and is characterized by high TC, TG, and LDL-C and low HDL-C levels [6]. LDL-C and other ApoB-rich lipoproteins accumulate in the arterial wall, playing a key role in atherosclerotic plaque formation and subsequent related cardiovascular events [19]. Studies have shown that the risk of developing carotid plaque increases by 62% for every 1 mM increase in serum LDL-C concentration [20]. Lipid levels in the plasma are influenced by many factors, and genetic variation in lipid-related genes leads to diverse phenotypes in different countries and ethnicities. Previous GWASs demonstrated associations between the *TUBB* and *WWOX* genes and LDL-C and HDL-C levels, and differences in the expression of these two genes in different populations contributes to diversity in lipid traits. According to recently updated data from 1000Genomes (http://www.ncbi.nlm.nih.gov/snp/), alternate allele frequencies in American, European, East Asian, and African populations, respectively, are as follows: *TUBB* rs3132584T, 0.1800, 0.1769, 0.1796, and 0.4803; *TUBB* rs3130685T, 0.5320, 0.4632, 0.5367, and 0.5479; *WWOX* rs2222896G, 0.5750, 0.6252, 0.6131, and 0.8313; and *WWOX* rs2548861G, 0.6341, 0.5656, 0.6282, and 0.2829. In this study, the frequencies of these allelic mutations in the Maonan population differed significantly from their frequencies in the CHB population: frequencies of the rs3132584T, rs3130685T, rs2222896G, and rs2548861G alleles in the Maonan and the CHB populations, respectively, were 0.2168 *vs.* 0.1829, 0.5730 *vs.* 0.5444, 0.5607 *vs.* 0.6341, and 0.6184 *vs.* 0.5488. Moreover, the frequency of the mutant homozygous TT genotype at rs3132584 was 0.0471 in the Maonan population but 0 in the CHB population, further demonstrating the differences in gene distribution between these two ethnic groups. Furthermore, although genotypic frequencies for rs3132584, rs3130685, and rs2222896 in the Maonan population differed significantly between the normal and dyslipidemia groups, only rs2222896 genotype was correlated with various serum lipid parameters. Normal group subjects with the rs2222896GG genotype had higher LDL-C and lower HDL-C and ApoA1 concentrations than those with other genotypes. This association between rs2222896 and serum LDL-C level was consistent with a previous study [21]. Additionally, Yamada et al. identified two novel loci in *TUBB*, rs3132584 and rs3130685, that were associated with high LDL-C in an exome-wide association study of early-onset dyslipidemia [13]. Another study of 2911 Japanese subjects revealed that the reference alleles rs3132584A and rs3130685C were associated with high LDL-C (*P* < 0.0001). In this study, we found that a rs3132584 mutation from G to T was present at a higher frequency. In the dyslipidemia group, rs3132584G was associated with high LDL-C levels; however, we observed little association between rs3130685C and LDL-C concentration. Two previous studies [14, 22] indicated that the rs2548861 SNP in *WWOX* was associated with HDL-C concentration in the Young Finns Study population (*n* = 6,728, *P* = 6.9 × 10^-7^), but another [23] failed to replicate this result in the Spanish population (*n* = 801). In our study, an association between rs2548861 and HDL-C concentration was observed in the dyslipidemia group (*n* = 1129, *P* = 0.001) but not in the normal group (*n* = 864, *P* = 0.597). We also found that HDL-C concentration in the dyslipidemia group might be affected by other non-genetic factors. Thus, we speculated that mutations at rs2548861 were not correlated with HDL-C concentration in these subjects. A correlation analysis between genetic models and disease risk was then conducted to understand the relationship between alleles at these four SNP sites and dyslipidemia. We found that codominant, dominant, recessive, and log-additive models of the rs3132584, rs3130685, and rs2222896 SNPs identified significant differences in dyslipidemia. Using the log-additive models, we identified the direction of the associations between each minor allele frequency (MAF) of these three SNPs and dyslipidemia. The rs3132584T and rs2222896A alleles were potential protective factors, while rs3130685C was a risk factor, for dyslipidemia. Various lipid-related SNPs and the frequencies of different alleles in the *TUBB* and *WWOX* genes can therefore lead to different clinical lipid phenotypes in different populations.

Genetic mechanisms are influenced by many types of interactions [24–26], including G × G and G × E interactions, which can have synergistic or contrasting effects on gene expression [27, 28]. Interactions among genes can include SNP-SNP interactions between individual genes, haplotype-haplotype interactions, and the combined effects of mutations in genes on different chromosomes [29, 30]. Detailed information on the four target SNPs was obtained from the 1000 Genome database. It indicated that there was a large gap between rs3132584 and rs3130685 at chromosomal positions 30720650 and 31238429, respectively, that prevented LD between them. In contrast, rs2222896 and rs2548861 at chromosomal positions 78058601 and 78624496, respectively, were about 500 kb from each other, resulting in a weak LD between them. Of the four common haplotypes observed for rs2222896 and rs2548861 alleles, rs2222896A-rs2548861T and rs2222896A-rs2548861G were associated with reduced risk of dyslipidemia while rs2222896G-rs2548861G and rs2222896G-rs2548861T were associated with elevated risk for dyslipidemia. In addition, multi-site mutations in *TUBB* and *WWOX* altered the incidence of dyslipidemia in the Maonan population. Thirteen types of multi-site mutation combinations for the two genes were screened using SHEsisPlus online software (http://shesisplus.bio-x.cn/SHEsis.html). Among these 13 combinations, rs3132584T-rs3130685T-rs2222896G-rs2548861T increased the risk of dyslipidemia 1.371-fold; all of the remaining 12 combinations could potentially decrease the risk of dyslipidemia. Although *TUBB* and *WWOX* are located on two different chromosomes, interaction models were successfully screened using the GMDR method (https://sourceforge.net/projects/gmdr/), and the effects of G × G and G × E interactions on dyslipidemia were examined further. The fourteen highest-performing models of interactions linked to dyslipidemia were identified; all had a 10 of 10 CV, training and testing balanced accuracy values greater than 0.5, and sign and permutation test *P* values less than 0.05. Furthermore, an intuitive interactive dendrogram and logistic regression analysis indicated that the rs3130685-rs2548861, rs3130685-rs2222896, H1-H3, I4-I8, and I4-110 interactions exhibited strong synergistic effects increasing the risk of dyslipidemia, while the rs3132584-rs2222896 and I1-I11 interactions decreased the risk of dyslipidemia. This study therefore demonstrated that *WWOX* haplotype and the combined effects of *TUBB* × *WWOX* have larger impacts on serum lipid levels and dyslipidemia than single SNPs in those genes [31]; additional experiments with larger samples sizes are needed to confirm this finding.

Many environmental factors, including aging, sex, unhealthy lifestyle (high-fat diet [32, 33], smoking, drinking), high blood glucose, and weight and obesity, are directly or indirectly involved in the pathogenesis of dyslipidemia. Moreover, these environmental factors can interact with lipid-related genes to alter lipid expression profiles [34, 35]. Several studies demonstrate that the incidence of hyperlipidemia is higher in elderly people (1259/1657, 76%, mean age 69 years) than in young people (203/494, 41%, mean age 29 years); this difference was attributed to age-associated declines in ribosome coverage in the vicinity of start codons and increases near stop codons, alterations in expression of genes associated with lipid metabolism, loss of hepatic LDL receptors, and decreases in sex hormone levels [36, 37]. Furthermore, the risk of dyslipidemia was higher in postmenopausal women and elderly men [38, 39]; food pickling processes can increase concentrations of secondary metabolites in lemons and onions, thus endangering health [40]; and heavy (8 or more drinks per week for women or 15 or more drinks per week for men) rather than low or moderate alcohol consumption was also linked to dangerous increases in lipid levels [41]. The Maonan ethnic group is located mainly in the Huanjiang region, which is known as the “hometown of cattle and grain” due to the prevalence of agriculture and beef cattle industries. Beef, duck, half-cooked chicken, animal offal, and pickled sour pork, snails, and vegetables, are common dishes in this region. In addition, consumption of wine made from rice, corn, sweet potatoes, pumpkins, etc., three times per day with meals and smoking cigarettes made from local dried tobacco leaves is common among Maonan men. Many high-risk environmental factors may therefore affect the Maonan population. We used random stratified sampling in our examination of dyslipidemia risk in this population to eliminate systematic differences in age and gender structure between the two groups. We found no differences in either proportion of subjects > 65 years old or severity of smoking between subgroups, but severity of alcohol consumption, BMI > 24 kg/m^2^, and FBS ≥ 7.0 mM were significantly higher in the dyslipidemia group than in the normal group, and these factors interacted with the *TUBB* and *WWOX* SNPs to affect the prevalence of dyslipidemia. Pairwise multiple regression interaction models and optimal interaction models from GMDR revealed that the rs2222896G-rs2548861G haplotype interacted with smoking, overweight/obesity, or FBS ≥ 7.0 mM, while the rs2222896G-rs2548861T haplotype interacted with old age, female sex, smoking, FBS ≥ 7.0 mM, or overweight status/obesity to exacerbate the risk of dyslipidemia. In addition, age > 65 years increased the synergistic effect of rs2222896-rs3130685, and FBS ≥ 7.0 mM increased the synergistic effects of H1-H3 or rs2222896-rs3132584 on dyslipidemia. In contrast, interactions between the rs2222896A-rs2548861T haplotype and female sex, old age, smoking, and drinking, and between the rs2222896A-rs2548861G haplotype and overweight status/obesity or FBS ≥ 7.0 mM, decreased the risk of dyslipidemia. Finally, overweight status/obesity decreased the protective effects of H1-H3 or I1-I11 interactions against dyslipidemia, and drinking weakened the increased risk of dyslipidemia associated with rs2222896, possibly because most subjects were low-moderate drinkers.

Blood pressure is also modified by multiple genetic and environmental factors and their interactions [42–44], and dyslipidemia is a common independent risk for hypertension [45]. We therefore further explored differences in the incidence of hypertension between the two groups and found that the prevalence of hypertension was associated with gender, age, BMI, FBS, smoking, drinking, and dyslipidemia. Interestingly, we also found that the rs3132584 and rs2222896 SNP interaction that affected risk of dyslipidemia also significantly increased the risk of hypertension; this might be a novel explanation of how dyslipidemia contributes to the pathogenesis of hypertension.

Some important strengths and weaknesses of this study should be considered when interpreting the results. Notable strengths include the following: (1) identification of differences in *TUBB* and *WWOX* mutation frequencies between the Maonan ethnic group and the CHB population provides novel data for human genomic studies; (2) detection of *TUBB-WWOX* interactions and G × E interactions that affected the prevalence of dyslipidemia might provide new therapeutic targets for dyslipidemia; and (3) this study characterized an integrated effect of the *TUBB* rs3132584 and *WWOX* rs2222896 SNPs on dyslipidemia that could also predict the risk of hypertension, identifying a possible novel pathogenic mechanism by which dyslipidemia can lead to hypertension. However, the following limitations should also be considered: (1) this study did not include other variable risk factors, such as daily exercise habits and anti-hyperlipidemia interventions; (2) despite the unique diet (high salt and sour, high fat and alcohol) associated with the Maonan population, intake of these foods was not accurately characterized and their effects on dyslipidemia could not be examined; (3) larger sample sizes are needed to verify the results of this study; and (4) biological function studies are needed to further examine the effects of interactions between these two genes on dyslipidemia.

In summary, this study identified associations between *TUBB*, *WWOX*, environmental factors, serum lipid levels, and dyslipidemia in the Maonan population. Our findings revealed that, compared to single-locus effects, *TUBB-WWOX*-environment interactions can result in synergistic or contrasting effects on incidence of dyslipidemia, thereby increasing or reducing the risk of dyslipidemia.

## MATERIALS AND METHODS

### Participants

A total of 1993 unrelated subjects were selected from our previous sample library in 2015 through stratified random sampling [46]; all subjects were from three generations of the Maonan ethic group and lived in the Maonan Autonomous County of Huanjiang in Southern China. Subjects were separated into normal lipid and dyslipidemia groups with similar age and gender distributions. The mean age of the 864 normal group subjects was 55.31 ± 13.08 years; 431 (49.88%) were males and 433 (50.12%) were females. The mean age of the 1129 dyslipidemia group subjects was 56.04 ± 12.88 years; 545 (48.27%) were males and 584 (51.73%) were females. All subjects were generally healthy without a history of diabetes, thyroid disease, autoimmune disease, cardiovascular disease, stroke, renal disease, tumor, or mental illness. The participants did not take medicine or eat foods with known lipid-lowering properties. The study was reviewed and approved by the Ethics Committee of the First Affiliated Hospital, Guangxi Medical University (No. Lunshen 2014-KY-Guoji-001; March 7, 2014). Subjects who met inclusion criteria and signed informed consent were enrolled after professional investigators explained the experimental rules.

### Laboratory values

Serum lipid levels were measured in peripheral blood (3 mL) collected in the morning after fasting for more than 8 hours using an autoanalyzer (type 7170A; Hitachi Ltd., Tokyo, Japan). Serum TC, TG, HDL-C, and LDL-C levels were determined using commercially available enzymatic assay kits, and serum ApoA1 and ApoB levels were determined by turbidimetric immuno-assay [46–48].

### Variable definitions

Dyslipidemia is defined by elevated levels of TC, LDL-C, and TG and decreased HDL-C levels [6]. In this study, normal ranges for serum TC, TG, HDL-C, LDL-C, ApoA1, and ApoB levels and ApoA1/ApoB ratio were 3.10−5.17 mM, 0.56−1.70 mM, 0.90−1.81 mM, 2.70−3.10 mM, 1.00−1.78 g/L, 0.63−1.14 g/L, and 1.00−2.50, respectively. Dyslipidemia was therefore defined by any of the following either alone or in combination: TC > 5.17 mM, TG > 1.7 mM, LDL-C > 3.10 mM, and/or HDL-C < 0.9 mM [49]. Blood pressure was measured after the subject had been sitting for at least 5 minutes. An average SBP ≥ 140 mmHg and/or DBP ≥ 90 mmHg for three measurements over two days was defined as hypertension [50]. BMI was determined by dividing weight (kilograms) by height (meters squared); overweight and obese categories in China [51] are defined as BMI > 24 kg/m^2^ and BMI ≥ 28 kg/m^2^, respectively.

### SNP selection

The basic principles of gene and SNP selection were as follows: (1) *TUBB* and *WWOX* were selected from previous GWASs examining lipid metabolism. (2) SNPs correlated with lipid levels were selected using Haploview (Broad Institute of MIT and Harvard, Cambridge, MA, USA version 4.2). (3) SNP information was obtained from NCBI dbSNP Build 132 (http://www.ncbi.nlm.nih.gov/snp/). (4) Only SNPs with MAF greater than 10% were included. (5) Correlations between the identified *TUBB* rs3132584 and rs3130685 SNPs and *WWOX* rs2222896 and rs2548861 SNPs and blood lipids were confirmed in previous studies [13, 14, 22, 23].

### DNA quality control and genotyping

We used a phenol-chloroform method to extract DNA from peripheral blood. DNA was stored at -80° C until 10 μL (10 ng/μL) of each sample was sent to the Department for Next-Generation Sequencing, Sangon Biotech Co., Ltd. (Shanghai, China). The absorbance of the DNA sample was measured at 260 nm and 280 nm using a Shimadzu UV-1601 spectrophotometer. An A260 nm/A280 nm ratio of = 1.8 indicates a pure DNA sample. SNP genotyping was performed on HiSeq XTen sequencers (Illumina, San Diego, CA, USA). The forward and reverse primers for amplification of the four SNPs via polymerase chain reaction were as follows: 5′-AGATCGGTGCCAAGGTAAGAAT-3′ and 5′-CAAACCCGAAGAGCCCTTTTACTA-3′ for rs3132584; 5′-TGTCAAATGCCATTCTTTAAACCTCATGT-3′ and 5′-TTGGGACACTTTACTCCTGAATC-3′ for rs3130685; 5′-AGCTAAAGCCTGTAGACCATCAACATA-3′ and 5′-TCCTCTTTAACACAGGTGAAATGCA-3′ for rs2222896; and 5′-CCAAACCCTAAATAATTGTCTGGATGTT-3′ and 5′-TCTGAATTTCTGCCAAGTTAGATG-3′ for rs2548861.

### Statistical analyses

SPSS software, version 25 (SPSS Inc., Chicago, IL, USA) was used to perform statistical analyses. Normally distributed data are presented as means ± standard deviation (SD) and differences between groups were identified using Student’s unpaired *t*-test and one-way analysis of variance (ANOVA). Non-normally distributed data are shown as interquartile ranges and medians and differences between groups were identified using Mann-Whitney nonparametric tests. Differences in qualitative variables, including different ratios and HWE between the two groups, were analyzed using Chi square tests. Associations between genotypes or haplotypes and continuous serum lipid level data were assessed by multivariable linear regression. Differences in serum lipid levels associated with genotypes and haplotypes were considered statistically significant at *P* < 0.0125, and G × G or G × E interactions were considered significant at *P* < 0.006 (corresponding to *P* < 0.05 after Bonferroni correction for four or eight independent tests). OR values and 95% CIs were calculated by multiple logistic regression after adjustment for stratified risk factors including age, sex, tobacco and alcohol consumption, BMI, and FBS. HWE, genotypic and haplotypic frequencies, and *D*′ and *R*^2^ values used to describe LD were calculated using SHEsisPlus online software. GMDR online software was used to screen optimal SNP-SNP, haplotype-haplotype, G × G, and G × E interaction models. All other data visualizations were generated using GraphPad Prism (version 8.0.0).
